# Limbal Epithelial Stem Cells in Review: Immune and Lymphangiogenic Privilege and Their Clinical Relevance

**DOI:** 10.3390/cells15010091

**Published:** 2026-01-05

**Authors:** Berbang Meshko, Thomas Volatier, Claus Cursiefen, Maria Notara

**Affiliations:** 1 Department of Ophthalmology, Faculty of Medicine and University Hospital Cologne, University of Cologne, 50923 Köln, Germany; 2 Cluster of Excellence for Aging Research (CECAD), Faculty of Medicine and University Hospital Cologne, University of Cologne, 50923 Köln, Germany; 3 Center for Molecular Medicine Cologne (CMMC), Faculty of Medicine and University Hospital Cologne, University of Cologne, 50923 Köln, Germany

**Keywords:** cornea, stem cells, angiogenesis, epithelium

## Abstract

**Highlights:**

**What are the main findings?**

- Limbal epithelial stem cells (LESCs) are central regulators of corneal immune and (lymph)angiogenic privilege, integrating niche-derived biochemical and mechanical cues to balance regeneration with avascularity.
- There are key stem cell markers that identify functionally distinct limbal cell subsets, with specialised epithelial cells supporting repair-associated angiogenic responses and matching stromal cells exerting strong anti-inflammatory and anti-(lymph)angiogenic effects.

**What are the implications of the main findings?**

- Loss or dysfunction of the limbal niche disrupts immune and vascular privilege, driving limbal stem cell deficiency (LSCD), chronic inflammation, neovascularization, and vision loss.
- Future LSCD therapies should combine epithelial regeneration with stabilization of immune and vascular privilege, leveraging stem cell populations or their paracrine signals to achieve durable ocular surface restoration.

**Abstract:**

The cornea maintains transparency by preserving immune and (lymph)angiogenic privilege through active suppression of inflammation and vascular invasion, a process centrally regulated by limbal epithelial stem cells (LESCs) located at the corneoscleral junction. Beyond renewing the corneal epithelium, LESCs maintain immune and vascular balance via extracellular matrix interactions and paracrine signalling, exerting predominantly anti-inflammatory and anti-(lymph)angiogenic effects in vivo. Disruption of the limbal niche by trauma, UV exposure, or genetic disorders such as aniridia leads to limbal stem cell deficiency (LSCD), chronic inflammation, loss of corneal avascularity, and vision loss. The identification of ABCB5 as a key LESC marker has clarified functional limbal subsets, highlighting ABCB5^+^ epithelial cells as mediators of repair, remodelling, and immune suppression, and positioning them as promising therapeutic targets for treatments that restore both epithelial integrity and corneal immune privilege.

## 1. Introduction

The cornea is the major refractive element of the eye. To properly focus light into the eye, the cornea must efficiently transmit light through itself by remaining transparent [1,2]. The refraction and transparency of the cornea is maintained through precise arrangement of collagen in the corneal stroma [3]. The stromal cells that maintain this collagen matrix also match their own refractive index to the cornea’s by regulating their cytoplasmic content [4].

Loss of corneal transparency is often a downstream consequence of two failures: breakdown of immune privilege and escape from (lymph)angiogenic privilege. Clinically, once afferent lymphatic routes and efferent blood vessels invade the cornea, antigen trafficking and leukocyte recruitment accelerate, rejection risk rises in case of subsequent corneas transplantation, and vision declines, explaining why the cornea’s exceptional graft success has long been linked to its immune-privileged status and avascularity [5,6].

This is a dynamic balance maintained by reduced antigen presentation, FasL-mediated deletion of infiltrating effectors, anterior chamber-associated immune deviation ACAID, and endogenous vascular inhibitors, including soluble VEGF receptors (sVEGFR-2/-3) that neutralize VEGF-C/-D to suppress lymphangiogenesis, thereby limiting both edema-inducing lymphatic flux and the priming arm of alloimmunity. When this network is perturbed by inflammation, hypoxia, or injury, the cornea’s angiogenic balance is disrupted, allowing neovessels to breach the limbal barrier [7,8,9,10].

The region where this balance physically evident and most relevant is the limbus, an oblique boundary where the cornea meets the sclera that encircles the entire cornea [11]. At the limbus, LESCs occupy a niche adjacent to dense vascular and immune circuits yet normally sustain an avascular, immune-regulated surface. In this context, several putative LESC markers, including ABCB5, have been associated with regenerative potential and with modulation of the local microenvironment. Available evidence suggests that LESC integrate extracellular matrix and mechanotransductive cues, as well as paracrine signaling programs, that collectively influence leukocyte activity and vascular growth [12,13]. This review synthesizes the following key features of the corneal limbus: 1—how LESCs and their microenvironment preserve immune and lymphangiogenic privilege in health; 2—how common sources of corneal pathological stress (e.g., UV, inflammation) lead to LSCD and neovascularization; and 3—the emerging relevance of ABCB5-expressing cell populations in corneal repair.

## 2. Cornea Anatomy

**Figure 1:**
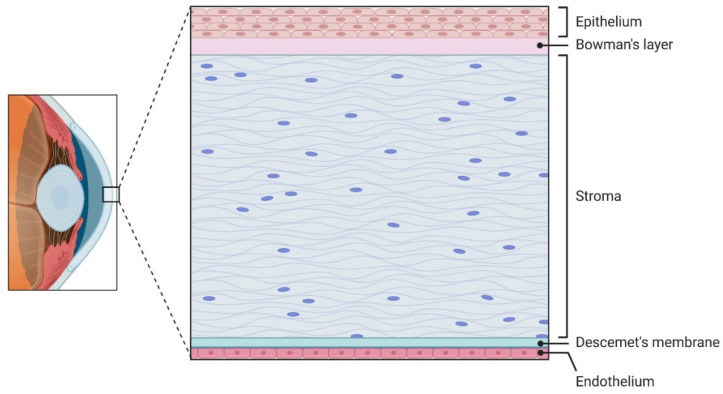
Cross-section of the corneal tissue layers, specifically in the central cornea. Stratified epithelium sits atop the acellular Bowman’s layer. The stroma makes up the bulk of the corneal volume with its highly organized collagen ECM. The acellular Descemet’s membrane separates the stroma from the innermost cell layer, the single-cell thick endothelium.

The cornea is the clear, dome-shaped front part of the eye. It contributes the majority of the eye’s total refractive power, making its shape and clarity critical for sharp vision. Anatomically, the human cornea consists of five layers, each distinct in its function for the maintenance of optical clarity and mechanical integrity (Figure 1). The epithelium consists of 5 to 7 layers of cells in humans and 2 to 3 layers in mice [14,15]. It comprises non-keratinized stratified squamous cells and acts as a physical barrier against environmental pathogens and trauma. Also, the epithelium contributes to the total corneal refractive power by maintaining a smooth optical surface. This layer undergoes continuous regeneration driven by LESC, which proliferate and migrate centripetally before differentiating and being shed from the surface. Beneath the epithelium, Bowman’s layer, which is an acellular, non-regenerating collagenous matrix situated between the epithelial basement membrane and the anterior stroma. In the central human cornea, Bowman’s layer measures approximately 10 μm in thickness and consists of randomly oriented 20 nm collagen fibrils with an undulating epithelial base. Scanning electron microscopy reveals a meshwork-like structure on its epithelial side, and it is clearly demarcated from the underlying substantia propria [16]. Functionally, Bowman’s layer contributes to corneal rigidity, tensile strength, and surface smoothness [17]. Furthermore, stromal nerve branches traverse this layer to form the sub-basal nerve plexus, located between Bowman’s layer and the basal epithelium, which extends fine perpendicular nerve terminals to innervate the anterior epithelial layers [18].

The corneal stroma makes up an estimated 80–85% of the total corneal thickness volume. It forms the primary structural scaffold of the cornea, and is essential for its transparency and mechanical strength [14]. The stroma consists of homogenous collagen fibrils of diameter 30 nm [19]. These fibrils are orthogonally arranged and organized into lamellae that run across the corneal surface. Collagen type I is the most abundant collagen type present, making up a reported 75% of the corneal collagen. Collagen type V is reported to be a consistent 20% of the total fibrillar collagen synthesized [20], and Collagen type III is reported to be present in quantities as low as 2%, but increases during wound healing and inflammation [21]. In the anterior stroma, the lamellae are thinner and more interwoven, providing enhanced resistance to shear forces and helping maintain anterior curvature. In contrast, the posterior stromal lamellae are thicker and more regularly parallel, contributing to overall tensile strength [22]. Interspersed between these lamellae are keratocytes, specialized fibroblast-like cells that maintain the extracellular matrix by secreting collagen and glycosaminoglycans (GAGs). Keratocytes also perform essential roles in wound healing responses [14]. The stroma is rich in GAGs, particularly keratan sulfate, chondroitin sulfate, and dermatan sulfate, which regulate hydration and precise interfibrillar spacing, both are critical features for the maintenance of the corneal shape and volume [22,23].

**Figure 2:**
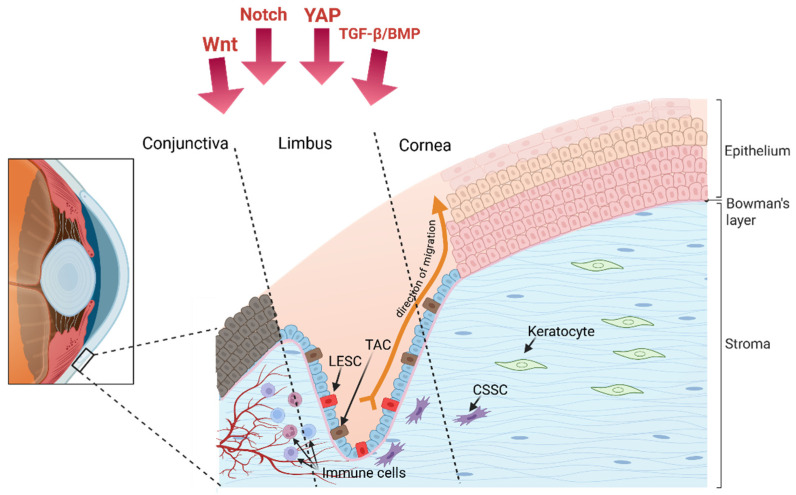
The limbus as a stem cell niche, with the Limbal Epithelial Stem Cell (LESC) and its descendent Transit-Amplifying Cell (TAC) that divide and migrate away from the limbus and into the central to form the corneal epithelium. Adjacent to the epithelial cells are the Corneal Stromal Stem Cells (CSSC), these are the cells that divide and differentiate to maintain the stromal keratocyte population. Under the limbal crypt are the blood vessels, lymphatic vessels, and immune cells; the presence of each of these components is regulated by the production of several important growth factor, some of which are shown here as Wnt Notch, YAP, and TGF-β/BMP.

Furthermore, recent findings highlight the existence of corneal stromal stem cells (CSSCs depicted in Figure 2), especially concentrated in the anterior limbal stroma. CSSCs exhibit mesenchymal markers (e.g., CD73, CD90, CD105) and modulate keratocyte and immune cells activity through paracrine immunomodulatory and matrix-regulating signals supporting stromal homeostasis and transparency [24,25].

Beneath the stroma lies Descemet’s membrane (DM), a distinct, acellular basement membrane critical for corneal structure and transparency. It consists of three distinct zones: an interfacial matrix that is anchored to the stroma, an anterior banded layer (2–4 μm) formed during fetal development, and a posterior non-banded layer that develops after birth and thickens with age (~1 μm per decade) [17,26]. DM is primarily composed of collagen types IV and VIII (the latter forming a hexagonal lattice), as well as laminin, fibronectin, and various proteoglycans [17,27]. the DM is the support for endothelial cell adhesion, regulates corneal hydration, and maintains curvature and biomechanical stability [17,28].

The innermost layer of the cornea, the endothelium, is a single layer of hexagonal cells responsible for regulating stromal hydration through active ion transport mechanisms, often described as a pump-leak system [29,30]. By maintaining precise stromal hydration, the endothelium preserves corneal transparency and prevents swelling, which would otherwise impair vision. Human endothelial cells exhibit limited regenerative capacity, compensating for cell loss mainly via enlargement and migration rather than division [31]. Consequently, endothelial dysfunction is a leading indication for corneal transplantation in industrialized countries [32]. Recent advances focus on ex vivo expansion and cell injection therapies to restore endothelial function [33].

## 3. The Corneal Limbus

### 3.1. Limbal Anatomy

The limbus is a narrow, ring-shaped transition zone measuring approximately 1.5 to 2 mm in width, located at the corneoscleral junction and completely encircling the peripheral cornea [34,35]. Anteriorly, it is defined by the termination of Bowman’s layer, while posteriorly it corresponds to the peripheral edge of Descemet’s membrane and aligns with the scleral spur [36,37]. Structurally, the limbus serves as a bridge between the transparent, avascular cornea and the opaque, vascularized sclera. The corneal stroma transitions peripherally into the scleral stroma, creating a continuous connective tissue [34]. The limbal stroma blends with adjacent stromal tissues and contains radial fibrovascular ridges known as the palisades of Vogt [38,39]. These palisades are innervated and vascularized structures rich in melanocytes and antigen-presenting cells, forming a specialized microenvironment [40,41]. From its inner to outer aspects, the limbus is adjacent to several critical structures, including the trabecular meshwork, scleral spur, Schlemm’s canal, episclera, conjunctival stroma, and conjunctival epithelium [37]. Unlike the avascular cornea, the limbus is highly vascularized, receiving its blood supply primarily from the anterior ciliary arteries and containing a dense network of blood and lymphatic vessels that facilitate metabolic exchange and immune cell presence [37,40]. Importantly, the palisades of Vogt serve as the main niche for LESCs, and within and beneath them, limbal epithelial crypts form epithelial extensions particularly enriched in LESCs that maintain corneal epithelial renewal [34,38,42,43]. Collectively, these anatomical and functional features establish the limbus as an essential zone for ocular surface homeostasis. It should be noted that the palisades of Vogt are absent in some species, including mice, and are instead predominantly found in primates and pigs. This species-specific difference in limbal architecture complicates direct comparison between mouse models and the human limbal stem cell niche [44,45].

### 3.2. ECM Composition and Anatomical Characteristics of the Limbal Niche

The extracellular matrix (ECM) of the limbal niche is fundamental for maintaining limbal epithelial stem cell (LESC) function, providing both structural support and biochemical cues essential for stemness. Unlike the central cornea, the limbal epithelial basement membrane reportedly lacks the long form of type XII collagen but is enriched in type IV collagen as well as laminin isoforms α2 and β2, which improve cell adhesion and selective cell signalling within the niche [41,46]. Additional components, including vitronectin, fibronectin, and tenascin-C, further distinguish the limbal ECM from the corneal stroma [34,47]. Tenascin-C is specifically expressed in the limbus in the same region as ABCG2+/p63+ basal epithelial cell clusters, indicating a key role in modulating cell adhesion and maintaining an undifferentiated stem/progenitor cell state [41,48].

Fibronectin, another important ECM glycoprotein, contributes to cell adhesion, migration, and wound healing. In rabbit models, it has been shown to promote self-renewal of LESCs, suggesting a supportive role in niche dynamics, though human-specific evidence is still evolving.

Hyaluronan (HA), a high-molecular-weight glycosaminoglycan synthesized by hyaluronan synthases (HAS1, HAS2, HAS3), is a major constituent of the limbal ECM. In murine models, HA is localized almost exclusively to the limbus, absent in the central cornea, with weaker staining in the perilimbal conjunctiva [49,50]. Disruption or downregulation of HAS enzymes, particularly HAS2, leads to decreased epithelial stratification, altered basal cell morphology, delayed wound healing, and loss of CK15+ cells, reflecting HA’s essential role in maintaining LESC function and preventing conjunctivalization [50]. Following corneal injury, HA expression transiently expands into the central cornea, accompanying a progenitor-like phenotype to promote epithelial repair. In inflammation, limbal HA can also promote lymphangiogenesis [49,50]. Collectively, these specialized ECM components create a unique and highly regulated microenvironment that preserves the regenerative capacity of limbal stem cells and safeguards corneal clarity and integrity [41].

Tenascin-C, fibronectin, and hyaluronan (HA) in the limbal ECM support LESC stemness which in turn regulate immune and vascular responses. Tenascin-C limits endothelial cell migration, [51], fibronectin promotes an anti-inflammatory environment [52], and HA helps suppress local inflammation [53]. Together, they contribute to the cornea’s immune and (lymph)angiogenic privilege.

### 3.3. Structure and Cellular Components of the Limbal Niche

In humans, LESCs reside within specialized anatomical microenvironments, including the palisades of Vogt, limbal crypts, and limbal lacunae, forming a highly structured niche that protects them from mechanical and UV-induced stress while providing vascular, neural, and extracellular matrix (ECM) support [42,54,55,56]. The human central cornea lacks a resident stem cell population, reinforcing the limbus as the principal stem cell reservoir of regenerative capacity [57,58].

The limbal niche does not only offer structural shelter but also actively regulates LESC behavior through autocrine and paracrine signals, as well as mechanical cues and specific ECM interactions [59,60,61,62]. The migratory maintenance of the corneal epithelium can be described by the X-Y-Z hypothesis, which describes corneal epithelial renewal as occurring in along three axes. LESCs proliferate and generate transit-amplifying cells TACs in the X axis, which migrate centripetally along a Y axis to replace terminally differentiated corneal epithelial cells that are shed from the surface along a Z axis [63,64].

Transcriptome and proteome analyses confirm that these niche components contribute to a microenvironment that supports both symmetric and asymmetric division of LESCs [65,66,67].

The LESC niche is highly cellularly diverse: limbal melanocytes protect LESCs from UV-induced damage and regulate their quiescence; limbal stromal cells, including keratocytes and CSSCs, modulate ECM composition and secrete paracrine factors supporting LESC maintenance [68,69]. Immune cells, particularly dendritic cells and macrophages, are also found adjacent to limbal crypts, contributing to niche immune protection [70,71]. Neural elements within the limbal niche contribute to LESC regulation through trophic signalling and cytokine gradients [72]. The limbal region is supported by a capillary bed branching from the episcleral arterial circle, providing nourishment to the limbal population [73,74]. The metabolic activity of the central cornea is comparatively lower and doesn’t require the arterial supply and venous drainage of the limbus. Instead, metabolic requirements are met by the delivery of glucose and oxygen from the aqueous humour and tear fluid [75].

### 3.4. LESC Markers

LESCs are characterized by the expression of a specific set of molecular markers, distinguishing them from transit-amplifying cells (TACs) and terminally differentiated corneal epithelial cells (See Table 1). Classic limbal stem cell markers include nuclear p63α, particularly the N-terminally truncated ΔNp63α isoform, which plays a critical role in maintaining the proliferative potential of limbal stem cells and their ability to migrate into the cornea [76,77,78]. In the absence of an attached limbus, p63α is absent from the corneal epithelium [79].

CK14 and CK15 are co-expressed in the basal limbal epithelium and serve as markers of undifferentiated progenitor epithelial cells, often used to identify the limbal epithelial stem cell (LESC) population [80,81]. ABCG2, a member of the ATP-binding cassette transporter family, is a marker of clonogenic stem cells, functioning as a side population determinant and contributing to cellular protection [82]. PAX6 regulates corneal epithelial development by establishing limbal stem cell identity during development and regulating gene expression profile of LESCs to retain non-keratinized epithelial cell properties [83]. Haploinsufficiency in Pax6 disrupts clonal expansion, epithelial maturation, and K12 expression, leading to defects analogous to human aniridia-related keratopathy [84,85,86,87]. Ksander and colleagues identified ABCB5, an ATP-binding cassette transporter, as a key marker of LESCs. ABCB5^+^ cells in the limbus demonstrate high clonogenic potential and the ability to support long-term corneal epithelial regeneration, confirming their identity as stem cells [12]. Supporting this, Vattulainen et al. showed that ABCB5^+^/∆Np63α^+^ cells derived from pluripotent stem cells exhibit LESC-like characteristics and enhanced wound healing capacity [12,88]. The co-expression of ABCB5 with established markers such as ∆Np63α and CK15 further validates its role in identifying functional LESC populations.

Other cell surface markers proposed include CD200 and CD109. CD200 appears on a small quiescent population capable of holoclone formation, and CD109 associated with more proliferative progenitors. Both were co-expressed with ∆Np63α, though the functional hierarchy among these subpopulations remains unclear [58,89].

**Table 1:** Summary of selected molecular markers associated with human corneal limbal stem cells (LSCs). The table lists markers commonly used to characterize limbal epithelial stem cell populations, including proteins positively associated with stemness as well as markers that are downregulated or absent in LSC-rich regions and instead associated with epithelial differentiation. Marker type, biological role, and key literature references are provided to support interpretation and experimental use.

Marker	Type	Role	References
ΔNp63α	Transcription factor	Highly expressed in basal limbal epithelial cells	[79]
ABCG2	ATP-binding cassette transporter	Marker of small basal limbal cell clusters	[82,90]
ABCB5	ATP-binding cassette transporter	Marks a slowly cycling population with regenerative capacity	[12,88,91]
CK14	Cytokeratin (intermediate filament)	Expressed in undifferentiated epithelial cells in limbal basal layer.	[90,92]
CK15	Cytokeratin	Co-expressed with stem/progenitor populations in limbus; lower in differentiated cells.	[80,90]
Integrin α9	Cell adhesion receptor	Enriched in basal limbus; interacts with limbal ECM components	[90,93]
Integrin β1	Cell adhesion receptor	Basal limbal expression, linked to epithelial progenitor identity.	[94]
N-cadherin	Cell adhesion molecule	Adhesion-linked stemness marker identified in basal limbal cells	[95]
Frizzled-7 (Fzd7)	Wnt receptor	Associated with canonical and non-canonical Wnt signalling in LESC	[96]
PAX6	Transcription factor	Master regulator of ocular identity; expressed in limbal progenitors.	[83]
BCAM	Basal Cell Adhesion Molecule	Mediates adhesion to laminin, associated with basal progenitor cells	[97]
CD200	Cell surface receptor	Immunoregulatory molecule; associated with quiescent limbal stem	[58,89]
CD109	Cell surface receptor	GPI-anchored protein; regulates TGF-β signaling	[92]
CK32	Cytokeratin	Expressed by cells in the limbal crypts	[98]

### 3.5. LESC Regulatory Pathways

**Figure 3:**
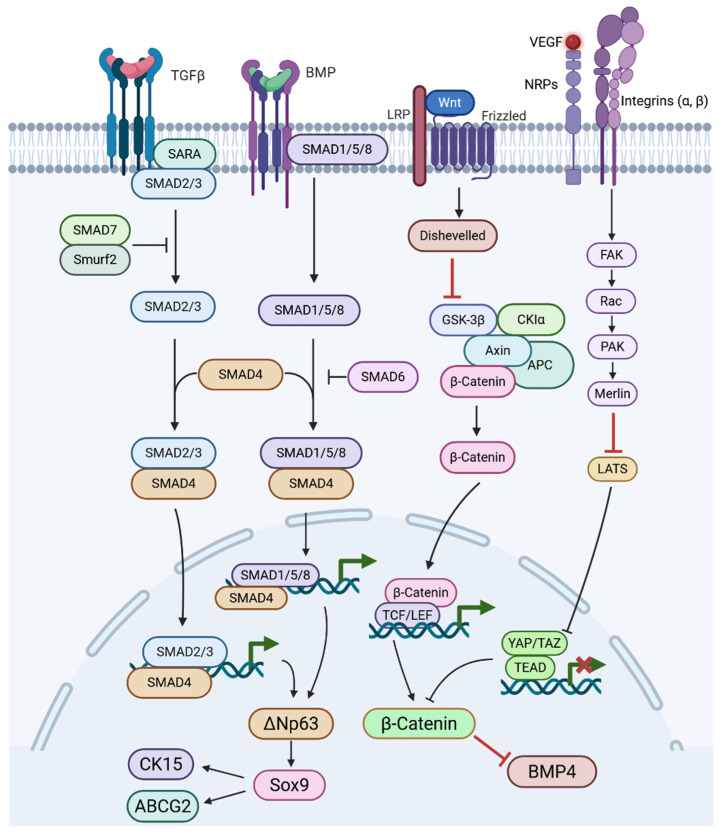
Diagram of Wnt, YAP, TGF-β, and BMP signalling that lead to the regulation of stem cell markers ΔNp63, Sox9, ABCG2, CK15, BMP4, and β-catenin. TGF-β binds its receptor, Smad2/3 are phosphorylated, the Smad complex enters the nucleus, and TGF-β/Smad activity regulates ΔNp63. BMP binds to type II BMP receptors and receptor-activated Smads (Smad1/5/8) are phosphorylated, Smad1/5/8 form a complex with Smad4 and translocate to the nucleus to regulates ΔNp63 in limbal epithelial stem cells. Wnt proteins bind to the Frizzled receptor and co-receptor LRP, this inhibits the β-catenin destruction complex, allowing β-catenin to enter the nucleus and partners with TCF/LEF transcription factors. Mechanical cues can prevent YAP translocation to the nucleus and allows β-Catenin, ABCG2, and CK15 to be highly expressed via ΔNp63 activation.

The healthy LESC niche is tightly regulated signalling cascades, including Wnt (canonical and non-canonical), Notch, TGF-β/BMP, Sonic hedgehog (SHH), and YAP/TAZ-Hippo, shape their fate (Figure 3). Activation or inhibition of these pathways is orchestrated through interactions with niche components such as extracellular matrix proteins, limbal mesenchymal cells, feeder cells, and human amniotic membrane (HAM) [99].

#### 3.5.1. Wnt Signalling

Canonical and non-canonical Wnt signalling play critical roles in LESC proliferation and maintenance through distinct yet interconnected mechanisms. This regulation is supported by Several Wnt ligands, Wnt2, Wnt6, Wnt11, and Wnt16b, and their receptors are more highly expressed in the limbus, where LESCs are located, suggesting region-specific regulation of Wnt activity [100,101]. Supporting the involvement of canonical signalling, β-catenin shows nuclear localization in basal limbal cells, where TCF4 is also co-expressed with stemness markers p63 and ABCG2 (Figure 3). Within this context, canonical Wnt signalling promotes LESC proliferation through the β-catenin/TCF4/survivin axis [101,102]. Activators like lithium chloride enhance colony formation [101,103], while inhibitors (e.g., IC15, XAV939) impair stemness and promote differentiation [104,105]. In parallel, non-canonical Wnt/PCP signalling, mediated by Wnt11 and Frizzled-7 (Fzd7), drives proliferation via syndecan-4/fibronectin/ROCK in rabbit and human LESCs [100,106]. MicroRNAs like miR-103/107 modulate both arms: they suppress canonical Wnt3a, activate PCP via JNK phosphorylation, and reduce YAP1 and Sox9, factors involved in stemness and Ca^2+^-dependent signalling [107]. Sox9, downstream of Wnt/Ca^2+^, can suppress β-catenin, indicating antagonism between pathways [108]. Moreover, the HC-HA/PTX3 peptide promotes LESC quiescence by activating Wnt/PCP and BMP pathways in limbal fibroblasts [109]. Thus, a dynamic balance between canonical and non-canonical Wnt pathways, modulated by niche cues, underlies LESC fate and regenerative potential.

#### 3.5.2. Notch Signalling

Notch signalling is crucial for regulating the asymmetric division of limbal stem cells (LESCs) and corneal epithelial stratification. Notch components are broadly expressed in the cornea and limbus [110,111]. The intermittent presence of NICD, HES1, and HEY1 in limbal tissue suggests that Notch activation occurs during corneal regeneration [104]. Genetic knock out or pharmacological inhibition of Notch enhances LESC traits while suppressing epithelial differentiation, suggesting its suppression supports stemness [104,112]. Studies showed that the Notch ligand form matters: immobilised Jagged1 activates Notch [113], while soluble Jagged1 inhibits it [114]. Also, Notch’s effects depend on crosstalk with Wnt, NF-κB, and YAP/TAZ pathways [112,115,116]. Altogether, evidence indicates that Notch signalling inhibition promotes LESC maintenance and proper corneal regeneration, though Notch’s role is nuanced and context-dependent.

#### 3.5.3. TGFβ/BMP Signalling

Transforming Growth Factor β/Bone Morphogenic Protein (TGFβ/BMP) signalling counteracts Wnt signalling and activates canonical pathways through ligand binding to type II receptors, which phosphorylate type I receptors, leading to Smad2/3 or Smad1/5/8 activation and nuclear translocation with Smad4 to drive gene expression [117]. TGFβ receptors are more highly expressed in the basal limbal epithelium than in the central cornea [118,119], and BMP4 is upregulated in the human limbus [120]. In vitro, BMP4 and phosphorylated Smad1/5/8 are enriched in LESCs cultured with limbal niche cells, while BMP inhibition activates Wnt/β-catenin signalling, increases colony-forming efficiency, and K12 expression, suggesting BMP-Wnt balance regulates LESC proliferation [105]. Human amniotic membrane (HAM), a substrate used to culture transplantable LESCs, provides TGFβ and other factors that promote proliferation and anti-inflammatory effects [121,122]. TGFβ1 in HAM may induce MMP-9, aiding ECM remodelling and epithelial outgrowth from the limbal explant [123,124]. However, excess TGFβ1 can trigger EMT in LESCs, which is counteracted by Smad7 [125].

#### 3.5.4. Sonic Hedgehog Signalling

SHH signalling promotes LESC proliferation and prevents their terminal differentiation. SHH is active in the limbal basal epithelium and signals through the Patched–Smoothened–Gli axis to upregulate cyclin D1, driving cell cycle progression [126]. In human and rabbit LESCs, SHH activation increases Sox9 expression, which suppresses both stemness and differentiation markers while promoting progenitor proliferation [108]. SHH inhibition reduces colony-forming efficiency and blocks the proliferative effect of a 44–amino acid peptide derived from pigment epithelium-derived factor (PEDF), which activates LESCs via SHH/Gli1/Gli3 signalling [127].

### 3.6. Mechanotransduction and ECM Regulation of LESCs

LESCs detect mechanical signals, such as blinking and tear flow, through mechanotransduction, where ECM-derived forces are converted into intracellular responses via integrins and adhesion complexes [128,129].

The soft niche environment suppresses YAP/TAZ nuclear localisation and drives LESC, proliferation, stratification, and β-catenin–driven stemness (Figure 3) [130,131]. In contrast, stiffer substrates promote nuclear YAP/TAZ and BMP4 activation, driving differentiation and migration. In vivo, cytoplasmic YAP predominates in the limbus, reflecting this mechanical regulation [132].

Chemical injury increases niche stiffness and depletes LESCs, while collagenase-induced softening can restore stem cell markers in vitro and in animal models [23,133]. Integrins (α3β1, α6β4) activate Wnt/β-catenin via integrin-linked kinase (ILK), supporting stemness [134]. N-cadherin also modulates β-catenin signalling in response to mechanical cues [95].

ECM proteins are major contributors of the limbus’ mechanical properties. Laminin-521 and Laminin-511 promote LESC proliferation through integrins, while Laminin-332 maintains undifferentiated markers [135]. Collagen synthesis (e.g., via ascorbic acid) enhances stemness independent of antioxidant effects [136,137]. Fibronectin activates non-canonical Wnt/PCP signalling via Frizzled-7 and Syndecan-4, enhancing self-renewal [106,138]. SPARC boosts ABCG2 and p63 through MAPK/JNK signalling, further reinforcing progenitor identity [139,140].

## 4. Corneal Privilege

LESCs are closely linked to both immune privilege and blood/lymphatic vessel privilege because they maintain the epithelial barrier and release factors that suppress inflammation and pathological vessel growth.

### 4.1. Immune Privilege

The cornea maintains its immune privilege through a combination of active and passive mechanisms that act across the three darts of an immune reflex arc [141,142]. Corneal avascularity limits immune cell trafficking and antigen exposure, thereby contributing to immune privilege while preserving the capacity for immune surveillance [143]. This avascularity is not a passive state but is actively sustained by anti-(lymph)angiogenic factors, ensuring that even after injury, such as refractive laser surgery, the cornea remains largely free of neovascularization [5].

Functionally, immune privilege is enhanced by reduced expression of major histocompatibility complex (MHC) class I molecules and a paucity of MHC class II-positive antigen-presenting cells within the corneal tissue [144]. This limited antigen presentation capacity dampens immune activation. In addition, the corneal epithelium expresses CD95 ligand (FasL), which can induce apoptosis in Fas-expressing immune cells, further contributing to immune tolerance [145,146]. Physiologically, anterior chamber-associated immune deviation (ACAID) induces systemic tolerance by generating regulatory T cells that suppress delayed-type hypersensitivity while preserving non-complement-fixing antibody production [147].

Beyond structural and cellular mechanisms, soluble immunomodulatory factors within the aqueous humour and corneal microenvironment, such as transforming growth factor-beta (TGF-β) and alpha-melanocyte-stimulating hormone (α-MSH), actively suppress inflammatory responses [148]. The interplay between these soluble mediators and structural barriers creates a tightly regulated microenvironment, where angiogenic and immune pathways are intricately linked. Notably, pro-angiogenic mediators like vascular endothelial growth factor (VEGF) have dual roles, influencing both vascular growth and immune cell recruitment [149].

### 4.2. Lymphangiogenic Privilege

**Figure 4:**
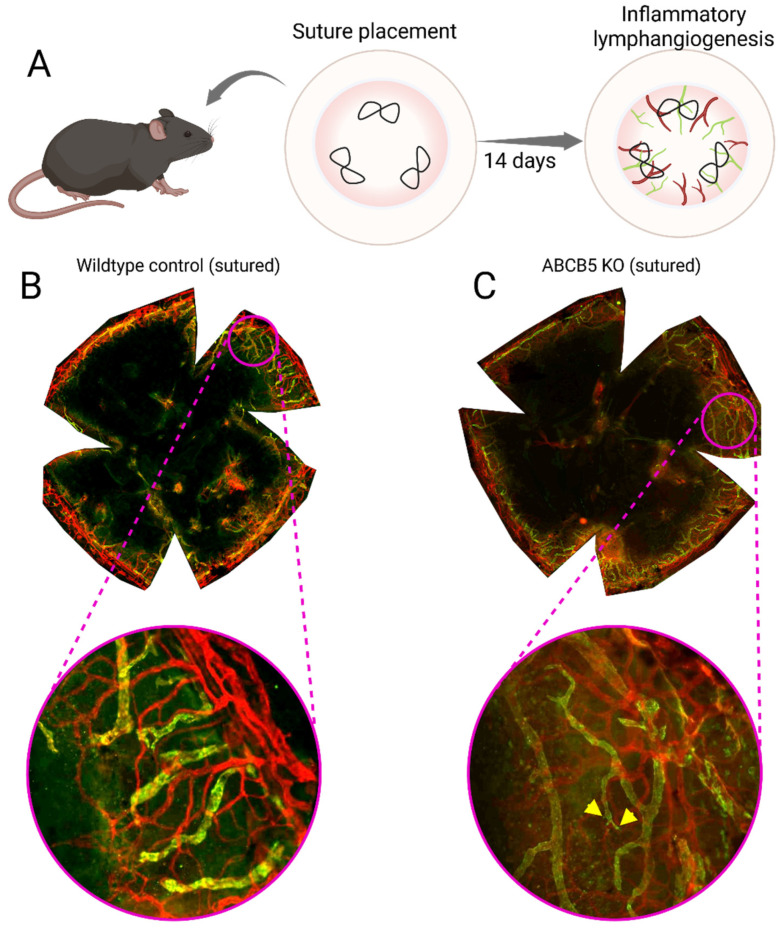
Mouse model of inflammatory lymphangiogenesis using three intrastromal, figure-eight sutures to induce chronic inflammation in the cornea over the course of 2 weeks (**A**). Flat mount of a wildtype sutured murine cornea (**B**); the central cornea is now vascularized after two weeks of chronic inflammation, with the vessels clearly extending across the limbus. Flat mount of a ACBC5 KO sutured murine cornea (**C**), the central cornea is vascularized and there is a greater amount of lymphatic vessel sprouts (labelled with yellow wedges) compared to the wildtype sutured animal [167], the vessels clearly traverse the limbus. Blood vessels are stained red and lymphatic vessels are stained green.

The cornea lymph-angiogenic privilege is essential for maintaining optical transparency and immune homeostasis. Owing to its normally vessel-free status, the cornea is a widely used in vivo model for studying the biology of angiogenesis and lymphangiogenesis [5,150,151]. Pathological vascularisation of the mouse cornea can be induced by placing intra-stromal sutures in the cornea. These sutures will cause a chronic inflammation that will drive vessel to invade into the normally avascular cornea (Figure 4).

Pathological vascularization in the cornea is largely driven by vascular endothelial growth factor (VEGF) family members, which activate VEGF receptors on endothelial cells of the limbal arcade [152,153]. Triggers such as inflammation and hypoxia increase VEGF release, but under physiological conditions, the cornea resists vascular invasion through a threshold-based buffering system capable of neutralizing low concentrations of proangiogenic factors [10].

A range of endogenous anti-angiogenic mediators supports the maintenance of this vessel-free state. The endogenous antiangiogenic factors that play a role in angiogenic privilege can be categorized into various groups: Endostatin and its analogs (including endostatin, arrests, and tumstatin), plasminogen/serine protease inhibitors (such as angiostatin and pigment epithelium-derived factor [PEDF]), thrombospondin-1 and -2 (TSP-1 and -2), as well as soluble VEGF receptors (sVEGFRs) [5,154,155,156,157,158]. Among these, TSP-1 is of particular importance in maintaining corneal avascularity [159]. In parallel, the corneal epithelium contributes actively through the expression of soluble VEGF receptors (sVEGFR-1/2/3) [8,9,154]. Functionally, sVEGFR-1 sequesters VEGF-A and additionally suppresses VEGFR-1/-2 signalling by heterodimerization (Ambati et al., 2006) [154], whereas sVEGFR-2 and sVEGFR-3 act as specific inhibitors of lymphangiogenesis by neutralizing VEGF-C and VEGF-D [8,9].

Further layers of inhibition derive from immunoregulatory factors in the aqueous humor, including α-MSH and VIP, which display both anti-angiogenic and anti-lymphangiogenic activity [160]. Under hypoxic conditions, VEGF-A, VEGF-C, and VEGF-D are normally upregulated [161]; however, the cornea counterbalances this with the expression of inhibitory PAS domain protein (IPAS), which antagonizes Hypoxia-inducible factor 1-alpha (HIF-1α) signalling [162]. Collectively, these overlapping mechanisms buffer minor pro-(lymph)angiogenic stimuli to maintain corneal transparencywhilst allowing neovascular response. While anti-hemangiogenic pathways are comparatively well defined, several endogenous inhibitors of lymphangiogenesis have also been identified in recent years, including by our group [159,163,164,165,166].

### 4.3. The Role of LESCs in Lymphangiogenic Privilege

Corneal cells play a pivotal role in sustaining the corneal lymph (angiogenic) privilege. Early studies demonstrated that differentiated corneal epithelium exerts strong anti-angiogenic properties [10,168,169,170]. Anatomically, the limbus represents the vascularized junction between the avascular cornea and the conjunctiva and functions as a physical barrier against the ingrowth of blood and lymphatic vessels. Within this specialized microenvironment, the limbal niche, a complex interplay of fibroblasts, melanocytes, immune cells, and a unique extracellular matrix enriched in collagen IV α1/α2 chains, laminin α5, β2, and γ1 chains, nidogen-1/-2, and thrombospondin-4, supports the maintenance of resident LESCs. These LESCs are located at the basal layers of the limbal epithelium, concentrated within stromal crypts of the palisades of Vogt [41], and provide the continuous renewal of the corneal epithelium, replacing cells shed through physiological turnover or injury [54].

The first identification of vascular endothelial growth factor (VEGF) in the cornea came from immunolocalization studies by van Setten, who described its presence in the basal epithelial layer [171]. Subsequent work in a rat model linked LESC deficiency to VEGF induction and inflammation, where VEGF upregulation coincided with corneal neovascularization [172]. Following injury, infiltrating leukocytes further contribute to VEGF secretion [173,174]. Specifically, VEGF-A165 and VEGF-A189 mRNA were strongly induced after corneal cautery, while VEGF transcripts localized to neutrophils and macrophages [173]. In addition, VEGF, TGF-α, and TGF-β1 were detected within the basal epithelium, endothelial cells of newly formed vessels, and infiltrating immune cells such as T lymphocytes and macrophages [143]. Despite this, the precise mechanisms by which the limbal niche, and in particular its stem cells, safeguard corneal avascularity remain incompletely understood. However, in LESC deficiency multiple soluble factors, including cytokines, chemokines, and growth factors, drive pathological neovascularization [175].

Addressing this question, Veréb and colleagues used genome-wide microarray profiling to compare ex vivo cultured putative LESCs with differentiated corneal epithelial cells. They showed that LESCs exhibited a more pro-angiogenic transcriptional profile [176]. Upregulated genes included Fibronectin, SERPINE1, MMP9, and F3, while anti-angiogenic regulators such as PLG, TIMP-1, FOXO4, and TGFBR1 were downregulated. Several cytokines and growth factors with strong angiogenic potential, IL-1β, CXCL10, TGF-β1, VEGF-A, IL-6, and IL-8, were significantly upregulated in LESCs, together with EDN1, EREG, and BMP2. In contrast, differentiated epithelial cells displayed a relatively anti-angiogenic profile, with downregulation of FGF1, IL-17F, TGF-β2, and c-Kit ligand [176].

These findings are consistent with our own results showing that UVA/UVB-induced LESC differentiation, marked by loss of stem cell marker expression and reduced clonogenic potential, led to downregulation of pro-angiogenic cytokines and acquisition of an anti-angiogenic paracrine effect on vascular endothelial cells [54,177,178].

The concept of LESCs displaying a relatively pro-angiogenic transcriptional profile is counterintuitive, particularly given their clinical application in transplantation for limbal stem cell deficiency (LSCD), a condition frequently associated with chronic inflammation and pathological neovascularization [179]. To reconcile this paradox, several groups have highlighted the immunoregulatory properties of LESCs within the damaged niche. These cells promote an anti-inflammatory microenvironment in the transplanted wound bed, thereby counteracting angiogenic drive.

In vitro, mouse LESCs dose-dependently suppressed lymphocyte proliferation and reduced pro-inflammatory cytokine release. In mixed lymphocyte reactions, they were more immunosuppressive than mesenchymal stem cells or natural regulatory T cells. They also showed higher expression of Fas ligand and the anti-apoptotic genes Mcl-1, XIAP, and survivin. Overall, freshly isolated adult LESCs display strong immunomodulatory and anti-apoptotic properties [180].

Such immunoregulatory activity indirectly contributes to the maintenance of corneal avascularity, since infiltrating leukocytes and macrophages are a major source of pro-angiogenic factors that drive hemangiogenesis and lymphangiogenesis [149].

### 4.4. The ABCB5^+^ Limbal Epithelial Cell Population: Dual Regulators of Corneal Angiogenesis and Immune Privilege

Our recent work using ABCB5 as a selective marker has provided further mechanistic insight. ABCB5 is a transmembrane ATP-binding cassette transporter that identifies LESCs and stromal progenitors, allowing their functional characterization and isolation. In our first study, we showed that ABCB5^+^ LESCs regulate both developmental and inflammatory (lymph)angiogenesis in the murine cornea, demonstrating their active participation in vessel remodeling under homeostatic and stress conditions [167]. These cells were capable of modulating endothelial cell behavior through paracrine mechanisms, suggesting that they contribute to a finely tuned balance between pro- and anti-angiogenic signalling in the limbal niche.

Notably, in a follow-up study, we identified a distinct stromal ABCB5^+^ population with strong anti-inflammatory and anti-(lymph)angiogenic properties. These stromal cells secreted factors that inhibited the proliferation, migration, and tube formation of blood and lymphatic endothelial cells in vitro, highlighting their role in maintaining corneal angiogenic privilege [25]. Notably, these stromal ABCB5^+^ cells also expressed immunomodulatory molecules, suggesting that their paracrine activity helps suppress inflammation-driven neovascularization. Together, these studies show that ABCB5^+^ cells in different compartments of the limbus have context-dependent, complementary functions: epithelial ABCB5^+^ LESCs can promote angiogenic processes when required for repair or development, whereas stromal ABCB5^+^ progenitors act to reinforce the anti-angiogenic and anti-lymphangiogenic microenvironment.

## 5. Tip of the Balance: Limbal Niche Disruption and Loss of Privilege

**Figure 5:**
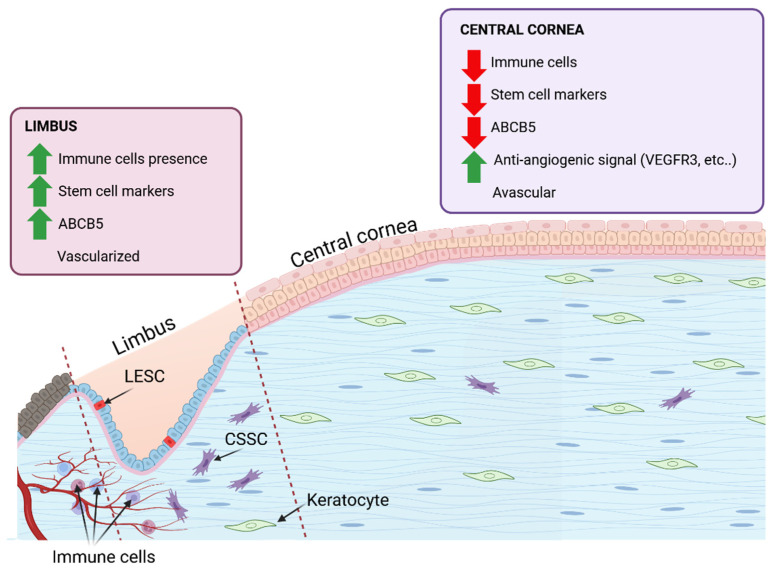
The corneal limbus and corneal centre provide different environmental cues. These cues influence the behaviour of the cells housed within and the overall tissue functionality. At the limbus, partial vascularisation provides greater oxygen and nutrient access, while the avascular central cornea depends on diffusion, both passive and active, for sustenance. The presence of immune cells at the limbus is partly mediated by the stem cells that immuno-interact. The lower presence of stem cells in the central cornea means a reduced immune cell interactivity, as long as there is no injury.

The corneal limbal niche delicately maintains a balance that preserves corneal transparency, avascularity, and regenerative capacity (Figure 5). Disruption of this equilibrium, through injury, genetic defects, or environmental stressors, can lead to LSCD, inflammation, and corneal (lymph)angiogenesis. Once this “tip of the balance” occurs, the cornea becomes susceptible to chronic epithelial defects, conjunctivalization, neovascularization, and severe vision impairment [35,54].

Lymphatic vessels contribute to immune surveillance and regulation in limbal region, particularly when they are recruited during injury or inflammation. Insights from skin reveal that the resident stem cells reshape the adjacent lymphatic vessels to better regulate their own activity [181]. There is a dynamic remodeling of the lymphatic vessels to support stem cells, with transient increases in lymphatic vessel caliber when stem cells are activated [182]. These findings underline the dynamic communication between stem cells and lymphatic system. In the cornea, lymphatic vessels similarly support limbal stem-cell function via Prox1-mediated paracrine signaling, and by draining inflammatory cytokines or immune cells that would otherwise impair stem cell maintenance [183].

### 5.1. LSCD Leading to Inflammation and Neovascularization

LSCD occurs when LESCs are depleted or their niche is severely compromised, resulting in impaired maintenance of the corneal epithelium. This promotes conjunctival epithelial ingrowth (conjunctivalization), persistent epithelial defects, chronic stromal inflammation, and neovascularization [184,185]. Loss of LESCs also permits pro-angiogenic factors such as VEGF-A to override anti-angiogenic mechanisms, further promoting vascular invasion into the normally avascular cornea [54,149].

Several conditions can lead to LSCD by severely damaging the LESCs or limbal niche [186]. These include ocular cicatricial pemphigoid (OCP), Stevens-Johnson syndrome (SJS), thermal or chemical burns, contact lens use, ultraviolet (UV) irradiation, multiple eye surgeries, the use of chemotherapeutic drugs like 5-FU (Fluorouracil) and MMC (Mitomycin C), and congenital aniridia [54,187,188].

### 5.2. Trauma

Severe ocular surface trauma, particularly chemical and thermal burns, is a leading cause of limbal stem cell deficiency (LSCD). Alkali burns rapidly penetrate ocular tissues, resulting in extensive loss of limbal stem cells, disruption of the stem cell niche, and persistent activation of inflammatory and angiogenic pathways [35,72,189]. This process triggers the release of cytokines, chemokines, and matrix metalloproteinases, which promote stromal remodelling, neovascularization, and conjunctivalisation [190]. In contrast, thermal burns induce similar pathological changes through protein denaturation, ischemic injury, and sustained inflammatory responses. These mechanisms compromise limbal barrier integrity and drive progressive corneal opacification [188,189]. Both types of burns eliminate the stem cell pool and destabilize the microenvironment required for epithelial regeneration, resulting in a chronic disease state characteristic of burn-induced LSCD.

### 5.3. Ultraviolet Irradiation in Limbal Stem Cell Deficiency

UV radiation directly damages ocular structures and can lead to partial or total blindness. The cornea is particularly susceptible due to its transparency and curvature, which amplify UV intensity up to 20-fold at the nasal limbus, where LESCs reside. This region is the site of pterygium, a noncancerous, often bilateral vascularized corneal growth that invades the limbal barrier, alters epithelial morphology, activates fibroblasts, and promotes inflammation, neovascularization, and extracellular matrix remodeling [54,151,191].

Short-term UVA and UVB exposure differentially affect the limbal niche. UVA irradiation reduces LESC marker expression and colony-forming efficiency while creating an anti-inflammatory and antilymphangiogenic micromilieu. In contrast, UVB triggers proinflammatory and macrophage-recruiting cytokines (TNFα, MCP1, IFN-γ), promoting immune cell infiltration, neovascularization, and niche dysfunction [177,178]. Limbal fibroblasts are key mediators in this process: UV-irradiated fibroblasts lose their ability to maintain LESC phenotype and alter paracrine signalling, shifting the balance between pro- and antiangiogenic responses depending on the type of UV exposure.

Mechanistic studies reinforce these findings: Repeated UVB exposure reduces ABCB5, a key stem cell marker, and activates epithelial–mesenchymal transition (EMT) and fibrosis, driving LESCs toward a scar-forming phenotype, as also observed in pterygium tissue [192]. Short bursts of UVB induce persistent DNA damage that only partially responds to repair mechanisms, highlighting the ongoing vulnerability of LESCs [193].

Clinically, pterygium acts as a visible example of UV-induced LSCD. This noncancerous, often bilateral, vascular growth invades the limbal barrier, alters the shape and behaviour of epithelial cells, activates fibroblasts, and results in increased inflammation, new vessel formation, and remodelling of surrounding tissue. Overall, these UV-driven mechanisms emphasise the importance of protective strategies, such as UV-blocking contact lenses, which laboratory studies have shown can help preserve stem cell health, reduce DNA damage, and maintain normal cell function [54,151,191].

### 5.4. Aniridia

Aniridia is a rare congenital eye disease with a prevalence of 1 in 40,000–100,000 births. Most cases result from heterozygous mutations in the PAX6 gene, inherited in an autosomal dominant pattern (≈70%) or arising as de novo mutations (≈30%), with occasional reports of paternal mosaicism [194,195]. Clinical features include iris hypoplasia, foveal hypoplasia, cataract, glaucoma, and aniridia-associated keratopathy (AAK), which leads to corneal conjunctivalization and neovascularization [194,196]. However, LESC proliferation is not fully lost, and stromal–epithelial interactions appear crucial in disease progression [86,197]. More recent studies show that niche dysfunction plays a central role, with loss of palisades of Vogt, basement membrane irregularities, inflammatory infiltration, extracellular matrix changes, abnormal melanocytes, and disturbed Wnt/β-catenin and retinoic acid signalling, leading to epithelial maldifferentiation and PAX6 mislocalization [198]. Also, recent paper showed that ARK corneas display fetal-like signalling with increased Wnt/β-catenin, SHH, and mTOR activity, alongside reduced Notch1 due to elevated inhibitors [199]. These findings suggest that both intrinsic PAX6-related defects and extrinsic niche breakdown drive AAK progression.

## 6. Perspective: Future Directions in LSCD Therapy with a Focus ABCB5^+^ Cells Role in Anti-Inflammatory and Anti-Angiogenic Strategies and Present Challenges

Building upon current therapeutic approaches, future directions in LSCD treatment increasingly emphasize strategies that not only restore epithelial integrity but also stabilize the underlying niche. Over the past three decades, surgical transplantation of limbal tissue, whether as autografts, allografts, or ex vivo expanded LESCs, has established proof-of-principle that regeneration of a functional ocular surface is possible [200,201,202]. Yet these interventions are constrained by major challenges: autologous grafts are not an option in bilateral disease, allografts require systemic immunosuppression with attendant risks, and the long-term persistence of transplanted cells remains uncertain. Evidence suggests that their benefit may instead rely on indirect effects, such as reactivating residual host stem cells or modulating the limbal microenvironment, rather than durable epithelial replacement [98]. 

Substrate selection has played a pivotal role in shaping therapeutic outcomes. Amniotic membrane provides not only a physical carrier but also intrinsic anti-inflammatory and anti-angiogenic effects. However, its biological variability and dependence on processing methods prompted a search for alternatives such as fibrin gels, temperature-responsive polymers, or synthetic scaffolds, which aim to deliver more standardized and reproducible culture systems [188]. At the same time, regulatory authorities have classified LESC-based interventions as advanced therapy medicinal products, underscoring the need to develop xenobiotic-free and GMP-compliant protocols to ensure both safety and reproducibility [179,188,203].

As these clinical and regulatory foundations were established, research has increasingly shifted toward understanding the pathophysiology of LSCD at the level of the niche. The failure to maintain corneal avascularity and immune privilege is now seen as central to disease progression. Loss of LESCs compromises the limbal barrier, resulting in conjunctivalisation, chronic inflammation, and hem- and lymphangiogenesis [54]. These insights have redirected attention toward strategies that combine epithelial regeneration with anti-inflammatory and anti-angiogenic modulation. In this regard, ABCB5^+^ epithelial and stromal subpopulations have emerged as particularly promising, since they not only retain clonogenic and regenerative potential but also exert immunomodulatory and anti-(lymph)angiogenic effects, suggesting a dual mechanism of action that stabilizes the niche while restoring epithelial integrity [25,179].

Parallel efforts have emphasized the importance of standardized potency assays capable of predicting therapeutic efficacy. Macrophage suppression has been identified as a critical functional marker, reflecting the central role of macrophages in orchestrating VEGF-driven angiogenesis and inflammatory cascades (Cursiefen et al., 2004; Sadeghi et al., 2024) [149,204]. Such assays not only bridge basic research with clinical translation but also align cell-based approaches with molecular therapies targeting VEGF and related angiogenic pathways already validated in corneal neovascular disease [205,206].

These developments point toward a future in which LSCD therapy moves beyond simple stem cell replacement. The integration of transplantation with adjunctive immunomodulatory and anti-angiogenic interventions offers a path to more durable restoration of ocular surface homeostasis. Protecting and reinforcing the limbal niche, whether through the direct action of transplanted stem cells, the immunoregulatory properties of specialized subpopulations, or pharmacological inhibition of proangiogenic signalling, is likely to prove essential for long-term success. In this integrative model, surgery, cell therapy, and targeted molecular modulation converge, offering not only epithelial repair but also stabilization of the corneal microenvironment. As highlighted across recent Publications in the field, the next generation of therapies will be defined by their ability to combine these complementary strategies, uniting regenerative approaches with immune and vascular control, to achieve lasting restoration of vision in patients with limb stem cell deficiency.

Experimental studies reveal the immune-regulating role of LESCs. When expanded ex vivo, LESCs express several genes linked to angiogenesis, including fibronectin, MMP9, IL-6, and VEGFA [176]. This profile raised questions about whether LESCs might paradoxically promote angiogenesis. However, when studied in their native context, they behave differently: freshly isolated cells suppress T-cell proliferation and dampen inflammatory cytokines, sometimes more strongly than mesenchymal stem cells or regulatory T cells [180]. By restraining immune activation, they indirectly reduce VEGF signalling and help preserve avascularity [149]. The contrast between culture and in vivo data highlights a key point: the function of LESCs depends on their microenvironment, and flexibility may be central to their physiological role.

The fragility of this system is evident when it is challenged. Ultraviolet radiation provides a striking example. Short-term UVA exposure weakens stemness but promotes an anti-inflammatory and antilymphangiogenic setting, whereas UVB induces cytokines such as TNFα, MCP-1, and IFN-γ, triggering immune infiltration, neovascularization, and fibrosis [177,178]. Repeated UVB stress also reduces ABCB5 expression and pushes cells toward epithelial–mesenchymal transition, changes linked to pterygium development [192,193]. Similar collapse of privilege follows chemical burns, autoimmune inflammation, or congenital defects like aniridia [189,198]. Taken together, these examples show how easily the limbal barrier can be tipped, and they reinforce the idea that maintaining privilege is an ongoing, active process.

The discovery of ABCB5 as a marker of limbal progenitors has helped to clarify the picture. Epithelial ABCB5^+^ cells can support developmental or injury-associated angiogenesis, suggesting a transient role in repair [12,167]. Stromal ABCB5^+^ cells, in contrast, secrete factors that strongly inhibit endothelial proliferation, migration, and tube formation, while also suppressing inflammatory pathways [25]. This points to a cooperative system: epithelial subsets allow controlled vascular responses during healing, whereas stromal subsets reinforce immune and lymphangiogenic privilege under steady conditions. It seems likely that the success of the niche lies in this division of labor, balancing the competing demands of regeneration and avascularity.

These insights carry obvious weight for therapy. In limbal stem cell deficiency, the breakdown of cornea immune and angiogenic privilege is as damaging as the loss of renewal. Conjunctival ingrowth, chronic inflammation, and neovascularization drive progressive vision loss [188]. Transplantation of limbal tissue or ex vivo expanded cells has shown that the surface can be resurfaced [200,201], but these approaches are limited by bilateral disease, reliance on systemic immunosuppression, and uncertain long-term survival of transplanted cells. It has been suggested that the therapeutic success of LESC transplantation may rely less on long-term engraftment of donor cells and more on their ability to influence the host environment through paracrine mechanisms [98]. From this perspective, treatments that fail to address the immune and vascular aspects of limbal niche dysfunction are unlikely to achieve lasting restoration.

ABCB5^+^ subsets provide a framework for moving forward. Stromal ABCB5^+^ cells, with their potent immunomodulatory and anti-(lymph)angiogenic activity, could be used either directly in cell therapy or through their secreted products. Epithelial ABCB5^+^ cells remain indispensable for restoring the epithelial barrier, but their benefit is likely greater when paired with stromal subsets that reinforce privilege. The combination of both populations offers a more holistic therapeutic concept, one that addresses not only the surface defect but also the microenvironment that sustains long-term clarity. This will also help to address the global unmet need for options to promote graft survival in vascularized high-risk transplants [207].

Looking ahead, several challenges remain. The molecular signals that determine whether LESCs adopt a regenerative or immunomodulatory role are still poorly defined. Pathways including Wnt, Notch, and TGFβ/BMP are implicated [101,105], but their hierarchy and interactions remain uncertain. Standardized tests, such as macrophage suppression or inhibition of lymphatic endothelial growth, could bridge laboratory findings with clinical outcomes [149,204]. Advances in biomaterials are another promising direction. Scaffolds enriched with laminin-511/521 or collagen IV support limbal stemness and may provide more physiological carriers for transplantation [23,135].

## 7. Conclusions

Altogether, the evolving view of LESCs highlights them as both regenerative and regulatory cells, capable of adapting to context and balancing the competing needs of healing and vascular restraint. The identification of two ABCB5^+^ subsets, in the epithelia and stroma, now offers tools to isolate and study these roles with greater precision. This suggests the future of LSCD therapy lies in integrated strategies that couple epithelial regeneration with reinforcement of immune and vascular privilege. Paracrine or molecule-based therapies represent a more feasible strategy for sustained ocular surface regeneration, offering superior standardization and delivery compared to cell- or scaffold-based approaches. Clinically, approaches that combine epithelial repair with restoration of immune and lymphangiogenic privilege, using cell-based or paracrine strategies, are likely to be required for durable treatment of LSCD.

## Data Availability

No new data were created or analyzed in this study. Data sharing is not applicable to this article.
